# A Survey of Telepractice in Speech-Language Pathology and Audiology in India

**DOI:** 10.5195/ijt.2017.6233

**Published:** 2017-11-20

**Authors:** HARITHA S. MOHAN, AYESHA ANJUM, PREMA K.S. RAO

**Affiliations:** 1 INDIA INSTITUTE OF SPEECH AND HEARING, MYSURU, KARNATAKA, INDIA

**Keywords:** Audiology, India, Speech Language Pathology, Telehealth, Telepractice

## Abstract

Telepractice has emerged as a form of service delivery to assess and treat individuals with communication disorders. The present study surveyed speech-language pathologists and audiologists in India about the use of telepractice. Two hundred and five (N=205) speech-language pathologists and audiologists responded to a questionnaire, with 12.19% reporting their use of telepractice to deliver clinical services. Respondents also indicated an urgent shortage of professionals in India to deliver clinical services in speech-language pathology and audiology, and opined that these needs can be met via the use of telepractice. India is well known throughout the world for the advanced application of Information and Communication Technology (ICT), with 931.95 million telephone subscribers, over 900 million mobile phone users, and the second-largest mobile phone usage in the world. India has also experienced a tremendous rise in the number of internet users. Therefore, India is well poised to fully develop telepractice to overcome the barriers of distance and amplify the availability of speech-language pathology, audiology and other healthcare services. But first, the widespread use of telepractice throughout the nation will require an improved infrastructure (e.g., to uphold privacy and security); training for professionals; and telepractice policies. While very promising, the deployment of telepractice throughout India will require the attention of policy makers and government organizations.

Innovation has led to technological advancements and the development of various forms of telecommunication systems. As a result of technological evolution, a large number of healthcare providers are employing telehealth. The term ‘telemedicine’ is used to describe the diagnostic and treatment services delivered over a distance by a physician, nurse, or related professional (Fong, Fong & Li, 2011).

Similarly, speech-language pathologists and audiologists are engaging in telepractice through use of online videoconferencing systems and other dedicated telecommunication systems to provide diagnostic and intervention services over geographical distances to a range of patients with communication disorders. Telepractice has been defined as “the application of telecommunications technology to deliver professional services at a distance by linking clinician to client, or clinician to clinician for assessment, intervention, and/or consultation” (ASHA, 2005a). Telepractice offers significant benefits, as it provides greater access to speech-language pathology and audiology services; improves access of services to clients with linguistic and cultural diversity; increases ease of collaboration among multi-disciplinary team members; and saves travel time with cost benefits for clients.

There is now considerable evidence to support the premise that service delivery through telepractice may actually enhance the quality of care provided, and, ultimately, quality of life (McCue, Fairman, & Pramuka, 2010; Winters & Winters, 2004). Winters and Winters (2004) suggest that telepractice has the potential to optimize the timing, intensity, and sequencing of intervention, leading to the greatest functional outcome for the client. Twenty-five percent of studies that assessed clients’ reactions to the use of telepractice reported favourable responses (Theodoros, 2011). It is also crucial to examine professionals’ attitudes toward the use of telepractice. The American Speech-Language-Hearing Association (ASHA, 2002) conducted a survey on use of telepractice among audiologists and speech-language pathologists. ASHA reported that 11% of respondents delivered services using telepractice, and 43% expressed interest in using it in the future.

India has a population of 1.34 billion (http://www.mospi.gov.in), with 1.8% of total population estimated to have a disability (Rehabilitation Council of India, 2016). In contrast, the Indian Speech and Hearing Association (ISHA) (2015) estimates there are 2500 qualified speech-language pathologists and audiologists in India. These data indicate a very low ratio of potential service receivers to available service providers. Strategies are needed to meet the service demands of persons in India with communication disorders.

On the positive side, India is well known throughout the world for the advanced application of Information and Communication Technology (ICT). India has 931.95 million telephone subscribers, over 900 million mobile phone users, and the second-largest mobile phone usage in the world. There has also been a tremendous rise in the number of internet users in India. At this juncture, telepractice use in India could overcome the barriers posed by distance and amplify the availability of speech and language services throughout the nation.

Additional research is required to determine if telepractice can be a viable form of service delivery in India. In contrast to Western countries, there is dearth of research evidence concerning telepractice in India. There is no reported data to account for the number of clinicians providing telepractice, the type of consultation services delivered, resource materials available, legal policies and guidelines for clients and clinicians, or training for telepractice service delivery. Hence there is a pressing need to examine the scope of telepractice in the India and the perspectives of clinicians.

The present study aims to explore telepractice in India. Speech-language pathologists and audiologists across the country were asked to complete an internet-based survey. Specifically, the study objectives were: (a) to report the status of telepractice in speech-language pathology and audiology in India; and (b) to report and compare the opinions of speech-language pathologists and audiologists about telepractice in India.

## METHOD

### PHASE I: DEVELOPMENT OF QUESTIONNAIRE

A questionnaire was developed to gather responses from two groups. Group I consisted of speech-language pathologists and audiologists engaged in telepractice. Group II included speech-language pathologists and audiologists not engaged in telepractice. The questionnaire was divided into two sections. The first section contained questions to collect demographic details of the participants in Groups I and II. The second section contained questions to elicit information from Group I about service delivery through telepractice. Twenty closed ended questions were included to study the perspectives of speech-language pathologists and audiologists on telepractice. Questions addressed four content areas: perceptions of professionals about service delivery through telepractice; characteristics of telepractice service; training and research in telepractice; and policies and guidelines for telepractice.

The questionnaire also included a sub-section with four questions for Group II on awareness of telepractice in speech-language pathology and audiology. Four additional mandatory questions were also included to elicit responses from Group I and Group II regarding the availability of telepractice providers in their regions; accessibility of resources for telepractice; and legal guidelines and policies in telepractice. The questionnaire was validated by five telepractitioners in speech-language pathology and audiology.

### PHASE II: ADMINISTRATION OF THE QUESTIONNAIRE

The sampling frame for the survey was approximately 2800 speech-language pathologists and audiologists registered with the Indian Speech and Hearing Association (ISHA). The questionnaire was configured with features of Google form and e-mailed to the registered ISHA members. All India Institute of Speech and Hearing Ethical Guidelines for Bio-Behavioural Research 2.1e and 2.4c were followed for the current study (Basavaraj, 2009). The e-mail included a letter of consent, objectives of the research, and the link to the survey questionnaire. Each participant was required to read an informed consent letter in which they agreed to the terms and conditions of the survey. Responses were collected over a period of three months, and kept confidential.

### PHASE III: ANALYSES OF RESPONSES

Participant responses were analysed by computing percentage values for the 20 questions. This was carried out separately for the overall responses of all participants (Groups I and II); for professionals engaged in telepractice (Group I); and professionals engaged in in-person service delivery (Group II). Further, a non-parametric statistical test (Test for equality of proportions) was employed for five questions (Q14, Q15, Q16, Q19, & Q20) to compare the opinions of professionals engaged in conventional in-person service delivery and service delivery through telepractice.

## RESULTS

### DEMOGRAPHICS

A total of 205 speech-language pathologists and audiologists completed the survey, a response rate of 7%. Responses were received from different states across India. Among the professionals, 48% (N=83)) were speech-language pathologists; 18.04% (N=37) were audiologists and 41.46% (N= 85) were speech-language pathologists and audiologists. All were engaged in providing clinical services. Group II included 87.80% (N=180) of the respondents who reported providing in-person service delivery. Approximately 12.19% (N=25) of the respondents reported using telepractice to deliver clinical services and were included in Group I.

**Figure 1:**
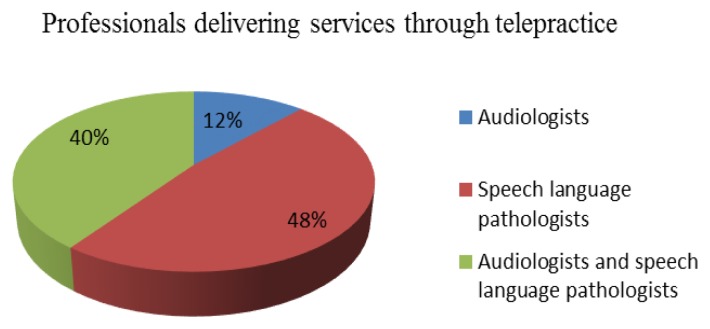
Professionals delivering services through telepractice (N= 205).

Among the professionals engaged in telepractice (Group I), speech-language pathologists were more likely to deliver speech and language therapy services than audiologists. Figure 1 depicts the percentage of professionals engaged in delivery of services through telepractice. Furthermore, 40% (N=10) provided both speech-language pathology and audiology services through telepractice.

### SURVEY FINDINGS

#### PERCEPTIONS OF PROFESSIONALS ENGAGED IN IN-PERSON SERVICE DELIVERY

The perceptions of speech-language pathologists and audiologists engaged in in-person service delivery (Group II) were examined in the following areas.

- Awareness of telepractice
- Viability of service delivery
- Requirement of certification for telepractice
- Infrastructure required

It was found that 55% (N=172) of the professionals were aware of service delivery through telepractice. Ninety percent (N= 162) of the respondents perceived that telepractice can be a viable form of service delivery; 73.3% (N=132) opined that the professional must be certified for telepractice; and 81.66% (N=147) stated that the infrastructure required for service delivery through telepractice is different from conventional in-person service delivery.

To an overwhelming degree, 92% (N =166) of participants were of the opinion that there is inadequate number of telepractice providers in their respective states in India. In addition, the survey revealed that 83.90% (N=151) of the professionals believed that sufficient resources are not available for telepractice. Most, 92.68% (N=167) were of the opinion that legal guidelines are needed for telepractice in India.

#### VIEWS OF TELEPRACTITIONERS IN SPEECH-LANGUAGE PATHOLOGY AND AUDIOLOGY

Only 12.19% (N=15) of the respondents reported using telepractice to deliver clinical services (Group I). Among the professionals engaged in telepractice, 48% (N=12) are speech-language pathologists and 12% (N=3) are audiologists. Within this group, 56% (N=14) of the professionals reported that they provide teletherapy services within India and 44% (N=11) provide services in other countries.

To compare opinions of Group I and Group II for the question regarding availability of telepractice providers in their respective states across India, the test for equality of proportions was carried out, using Smith’s Statistical Package, version 1.0. This statistical test determines the difference between the population proportions. The test results suggest no significant difference between the opinions of Group I and II concerning the unavailability of telepractice service providers in their respective states in India (0.120, p> .05, |z| 1.55).

### CHARACTERISTICS OF TELEPRACTITIONERS

#### SETTINGS OF SERVICE DELIVERY THROUGH TELEPRACTICE

**Figure 2:**
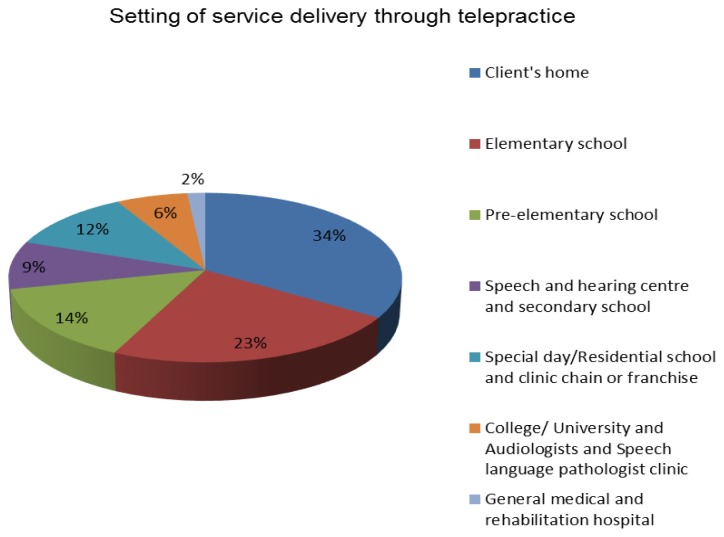
Settings of service delivery through telepractice.

Figure 2 represents percentages of the most common settings where clinicians provided tele-speech and teleaudiology services.

Results revealed that 36% (N=9) of telepractice providers were self-employed; 24% (N=6) were an employee of a public or a private non-profit organization; 20% (N=5) were an employee of a central government organization; and 4% (N=1) were an employee of a private organization.

### AGE GROUPS SERVED THROUGH TELEPRACTICE

Telepractice services were provided to clients throughout the lifespan. Table 1 shows the percentage of telepractitioners providing clinical services across various age groups.

### TYPES OF SERVICE DELIVERY

As shown in Table 2, telepractice service providers variously delivered screening, assessment, management, follow-up or monitoring/guidance and/or professional consultation.

### DISORDERS SERVED THROUGH TELEPRACTICE

Table 3 presents a summary of the disorder areas for which telepractice services were provided.

Results revealed that some disorders such as childhood aphasia, specific language impairment, motor speech disorders in children, dysphagia in children and adults, auditory processing disorders and vestibular disorders were managed via telepractice.

Fifty-six percent of the telepractitioners (N=14) reported they had received positive feedback from clients about telepractice services. In addition, 64% (N=16) of telepractitioners reported client satisfaction with follow-up/monitoring, and better access to services. Sixty percent (N= 15) reported client satisfaction concerning the regularity of telepractice services.

### TRAINING AND RESEARCH IN TELEPRACTICE

One hundred percent of respondents in Group I (N=12) and 28% (N=50) in Group II were of the opinion that a postgraduate degree in speech-language pathology or audiology is required to be qualified to deliver telepractice. In contrast, 28% (N=7) of respondents in Group I and 9.4% (N= 17) in Group II suggested that a short-term training certification course in telepractice should be made mandatory for professionals to deliver clinical services through telepractice.

Ninety-two percent (N=23) of respondents in Group I and 97.22% (N=175) in Group II, have not authored a publication on telepractice. This suggests a dearth of research on telepractice in speech-language pathology and audiology in India. Furthermore, 68% (N=17) of respondents in Group I and 86.11% (N=155) in Group II reported that there are currently insufficient resources (i.e., structural framework; technical support, materials) to provide appropriate speech-language pathology and audiology services in India.

### POLICIES AND GUIDELINES FOR TELEPRACTICE

A large number of respondents, 92% (N=23) in Group I and 92.77% (N=167) in Group II) were of the opinion that there should be mandatory legal guidelines for the conduct of telepractice in speech-language pathology and audiology. Eighty-eight percent (N=22) of respondents in Group I and 58.04% (N=119) in Group II opined that policies and technologies should protect clinician and client privacy and security in telepractice.

Finally, 72% (N= 18) of the respondents in Group I stated that a standard protocol to document the services delivered via telepractice is essential.

## DISCUSSION

This article surveyed the opinions of speech-language pathologists and audiologists about telepractice in the Indian sub-continent. Results suggest that 95.55% (N=172) of speech-language pathologists and audiologists who provide in-person service delivery (Group II) were aware that telepractice can be a form of service delivery. However, only 12.19% (N=25) of the survey respondents are engaged in telepractice (Group I) in India. This is similar to the findings of a 2002 survey of speech-language pathologists in the United States (ASHA, 2002). In the current study, among the professionals engaged in telepractice (Group I), speech-language pathologists were more likely than audiologists to engage in tele-speech therapy. Most of the professionals surveyed agreed that telepractice can be a viable form of service delivery to assess and treat persons with communication disorders.

Clients in India have limited access to speech-language and audiology services, as there is a shortage of service providers (Rao & Yashaswini, 2017). Respondents in the current survey expressed that there is an urgent need to meet the demands of the individuals seeking services in their respective states across India. This could be made possible if professionals could engage in telepractice to offer clinical services in speech-language pathology and audiology.

Furthermore, professionals were of the opinion that the infrastructure required for telepractice is significantly different from conventional service delivery. Newer technology enables clinicians to use laptops and tablets along with low-cost web-based videoconferencing systems. Technology has greatly enhanced the audio and video hardware and software that enables clinicians to offer quality services to their clients in a cost-effectiveness manner. When an adequate technological infrastructure is provided (e.g., to improve the likelihood of a trouble-free videoconferencing session) telepractice can be affordable, flexible, and reliable. For India, this points to a pressing need to lay norms for technical infrastructure for telepractice service delivery.

Based on the results of this survey, service providers in India have primarily learned to implement telepractice through personal experience rather than formal training. Most professionals engaged in telepractice in India were of the opinion that formal training and certification are necessary for professionals who employ this service delivery model. Systematic and comprehensive training in telepractice will be crucial as the use of telepractice increases in the future.

The privacy and security of both clients and clinicians are of critical importance in telepractice. Survey respondents recognized that use of open, unencrypted platforms poses inherent risks to privacy and security. The software in public domains that are largely in current use by telepractitioners in India poses a serious threat to the privacy security of both clinicians and clients. There is an urgent need to employ secured, licensed services while engaging in service delivery through telepractice. There is also a compelling need to put forth legal guidelines and policies for safe and secure service delivery.

There is the need to examine an even greater number of factors critical to telepractice service delivery in India. The current survey is limited in scope. First, the number of respondents is relatively small, although it is the representative of the relatively few professionals engaged in telepractice in India. Second, the survey did not collect any information on the infrastructure available to telepractitioners, the resource materials used, or the challenges faced by the telepractitioners during their sessions.

In spite of these limitations, this survey suggests an opportunity to examine, reform, and even create new guidelines for telepractice in India. And most significantly, it points to the pressing need for technological support and training to enable professionals to offer quality services. The insight gained from this survey offers directions to improve service delivery through telepractice in a country that already is well known throughout the world for the advanced application of Information and Communication Technology (ICT).

The findings of the survey endorse the feasibility of providing speech-language and audiology services through telepractice. The results reflect the opinion of the professionals for whom telepractice is a plausible mode of service delivery. The extensive technology available to healthcare providers such as speech-language pathologists and audiologists, and their clients, should be used to its fullest extent to enable better quality care in settings and regions where the physical presence of expertise is unavailable.

## Figures and Tables

**Table 1:** Age Range Served By Telepractice Service Providers

Age range	% of Respondents
Infants	12.97%
Paediatric	33.76%
Adolescents	14.28%
Adults	20.82%
Older adults	18.17%

**Table 2:** Services Delivered Through Telepractice

Age range	% of respondents
Screening	15.27%
Assessment	19.44%
Management	20.83%
Follow-up/monitoring guidance	27.77%
Professional consultation	16.69%

**Table 3:** Disorders Served Through Telepractice

Areas	% of respondents
Speech sound disorders in children	12.09%
Learning disability	9.67%
Childhood receptive and expressive language disorders	8.87%
Cognitive communication disorders	6.45%
Fluency disorders in adults	7.25%
Adult language disorders	5.64%
Voice and resonance disorders in adults	5.64%
Speech sound disorders in adults	5.64%
Autism spectrum disorders	4.83%
Accent modification	4.83%
Voice and resonance disorders in children	4.83%
Fluency disorders in children	4.83%
Aural rehabilitation	4.03%
Hearing aid or assistive technology maintenance	4.03%
Auditory verbal therapy	3.22%
Motor speech disorders	1.61%
Tinnitus	1.61%
Auditory processing disorders	0.80
Motor speech disorders in children	0.80%

## APPENDIX

A Survey of Telepractice in Speech-Language Pathology and Audiology in India

The objectives of the survey are,

- To study existing service delivery through telepractice in India
- To understand professionals’ views of on telepractice in speech-language pathology and audiology

*Indicates mandatory questions which cannot be skipped by the participants

### Informed consent*

I have been informed by the investigators about the study entitled “A survey of telepractice in Speech-Language pathology and audiology in India” I understand the purpose and procedures of the study. I declare that my participation in this study is entirely voluntary and that I may withdraw at any time without incurring a penalty. I do not have any financial or non-financial benefits from this study. I hereby give my consent to participate.

I Agree
I Disagree
Name*:
Age/Sex*:
Professional qualification*:
Date*:

### Instructions

Please answer the following questions pertaining to telepractice in speech-language pathology and audiology

1. In which state do you currently reside?*
2. Do you currently provide clinical services?*
  Yes
  No
3. In which specialty do you provide clinical services?
  Speech-Language Pathology
  Audiology
  Both
4. Do you provide clinical services in speech-Language pathology and/or audiology through telepractice?*
  Yes
  No
  If no,
  Are you aware of telepractice in speech-language pathology and/or audiology?
  Yes
  No
  Do you feel telepractice can be a viable form of service delivery?
  Yes
  No
  Do you think one has to be certified for telepractice?
  Yes
  No
  Do you feel infrastructure required for telepractice is same as face-to-face service delivery?
  Yes
  No
  If yes, respond to the questions below
5. To which state and/or country do you currently deliver telepractice services?
6. Which of the following best describes your employer?
  Self-employed (e.g., run own practice)
  Employee of a central or a state government organization
  Employee of a public or a private non-profit organization
  Others
7. In what type(s) of facilities are the clients you serve through telepractice? (select all that apply.)
  Pre-elementary (preschool)
  Elementary school
  Secondary school
  Special day/residential school
  Clients home
  College or university
  General medical hospital
  Rehabilitation hospital
  Private physician’s office
  Audiologist or Speech-Language pathologist’s clinic
  Speech and hearing center
  Clinic chain or franchise
  Others
8. What age groups do you regularly serve through telepractice? (select all that apply.) <6 months
  7 months – 2 years
  3 – 5 years
  6 – 11 years
  12 – 17 years
  18 – 64 years
  65 – 74 years
  >75 years
9. What type of services do you deliver through telepractice? (select all that apply.)
  Screening
  Assessment
  Management
  Follow-up/monitoring/guidance
  Professional consultation
  Others
10. In what area(s) do you provide services through telepractice? (select all that apply.)
  Accent modification (e.g., speech intelligibility in multi-linguals, second language learning, etc.,)
  Childhood receptive and expressive language disorder
  Childhood aphasia
  Learning disability
  Autism spectrum disorders
  Specific language impairment
  Voice and resonance disorders in children
  Speech sound disorder in children
  Fluency disorders in children
    Motor speech disorders in children (e.g., dysarthria, apraxia, etc.,)
    Dysphagia in children
  Adult language disorders (e.g., aphasia, dementia, etc.,)
  Cognitive communication disorders
  Voice and resonance disorders in adults
  Speech sound disorders in adults
  Fluency disorders in adults
  Motor speech disorders (e.g., dysarthria, apraxia, etc.,)
  Dysphagia in adults
  Auditory verbal therapy
  Aural rehabilitation
  Auditory processing disorders
  Hearing aid or assistive technology maintenance
  Vestibular disorders
  Tinnitus
  Others
11. Do you, or does your employer, receive payment for services delivered through telepractice?
  Yes
  No
  Uncertain
12. Which of the following qualification(s) do you feel is the most appropriate for telepractice? (select all that apply.)
  B.Sc. Speech-Language Pathology and Audiology
  M.Sc. Speech-Language Pathology/Audiology
  Diploma in Speech-Language and Hearing Sciences
  Short term certification course in telepractice
13. What benefits have your clients realized from services through telepractice?
  Regularity in service
  Satisfactory follow-up or monitoring
  Better access to service with respect to cost vs. benefit
14. In your opinion, are there adequate tele service providers in your state?*
  Yes
  No
15. Do you have any publications on telepractice in Speech-Language and/or audiology?*
  Yes
  No
16. Do you feel that the resources available are appropriate and sufficient for telepractice?*
  Yes
  No
17. Do you follow a standard protocol for documentation of your tele sessions?
  Yes
  No
18. What is the feedback offered by your clients about tele services?
  Very good
  Good
  Satisfactory
  Not satisfactory
19. Are legal guidelines required for telepractice?*
  Yes
  No
20. Do you feel that policies and technologies protect clinician and client privacy and security?*
  Yes
  No

Thank you for your participation!

## References

[b1-ijt-09-69] 29th Annual report 2015–16 of the Department of Empowerment of Persons with Disabilities, Rehabilitation Council of India, 2016.

[b2-ijt-09-69] American Speech-Language-Hearing Association (2005a). Knowledge and skills needed by speech-language pathologists providing clinical services via telepractice [Knowledge and Skills].

[b3-ijt-09-69] American Speech-Language-Hearing Association (2011). Survey of telepractice use among audiologists and speech-language pathologists, 2002.

[b4-ijt-09-69] Basavaraj V (2009). Ethical guidelines for bio-behavioural research.

[b5-ijt-09-69] Fong B, Fong ACM, Li CK (2011). Telemedicine technologies: Information technologies in medicine and telehealth.

[b6-ijt-09-69] Indian Speech and Hearing Association (2015). http://www.ishaindia.org.in/.

[b7-ijt-09-69] Indian Speech and Hearing Association Members on roll as of 12 May, 2017.

[b8-ijt-09-69] McCue M, Fairman A, Pramuka M (2010). Enhancing quality of life through telerehabilitation. Physical Medicine and Rehabilitation Clinics of North America.

[b9-ijt-09-69] NSSO (2002). GOI. Ministry of statistics and programme implementation, National Sample Survey Office. 2002.

[b10-ijt-09-69] Rao KS, Yashaswini R (2017). Tele service in speech-language pathology and audiology prospects and challenges.

[b11-ijt-09-69] Theodoros D (2011). Telepractice in speech-language pathology: The evidence, the challenges, and the future. Perspectives on Telepractice.

[b12-ijt-09-69] Winters JM, Winters JM (2004). A telehomecare model for optimizing rehabilitation outcomes. Telemedicine Journal & E-Health.

